# Cases of Neuralgia Spasmodica

**Published:** 1824-04

**Authors:** Benjamin Hutchinson


### Art. II.—*Cases of Neuralgia Spasmodica.* Communicated by Benjamin Hutchinson, Esq.

### *To the Editors of the Medical and Physical Journal.*

Gentlemen,—May I beg the favour of you to insert the following cases of Neuralgia in your ensuing Journal, and you will oblige your's, truly, B. HUTCHINSON.

*Copy of a Letter from* E. S. Simon Snow, Esq. *Surgeon, Chester.*

Sir,—I feel much pleasure in an opportunity of detailing to you the subjoined case, showing the decidedly good effects of the carbonate of iron, as recommended by you, in cases of tic douloureux. I intended to have sent this communication on the 16th instant; but afterwards conceived it would be better to wait to see if any recurrence of pain came on after the omission of the iron. I saw the young lady yesterday, and feel happy in saying that she continues quite well. I have the honour to be, Sir, with every sentiment of respect, your very obedient servant, E. S. SIMON SNOW, Surgeon.

Miss F., ætat. twenty-three, of slender and delicate constitution, was attacked, about two years ago, with great pain in the left side of the lower jaw, which was attributed to a carious tooth. The pain continued almost constantly, and the gum surrounding the tooth became so excessively tender, as to prevent her from masticating her food upon that side; and she could not, for a length of time, be prevailed upon to have the tooth extracted. Nostrums, of various kinds, were tried in vain to relieve it. In the course of last summer, she was recommended change of air; and for that purpose repaired to Flint, a very pleasant little town upon the river Dee. She remained there two or three months, without much remission of pain, which was occasionally so violent as to induce her to tie her bonnet over her chin as tight as possible, and afterwards used violent exercise, till, from fatigue, she fancied herself relieved. Soon after this time, the pain began to observe more regular paroxysms, which came on every evening, and continued from two to three hours.

The young lady's friends at length became very uneasy, and requested my attendance on the 28th of December last; when I found her suffering, according to her account, from a severe attack of tooth-ache. Upon examination of her mouth, I found one of the bicuspides much decayed, and the gum excessively irritable and ulcerated, attended with violent twitching pains shooting along the cheek to the neck and ear. I prescribed fomentations, and a strong embrocation of ether, camphor, and opium, to be freely applied to the gum, and within the cheek; and five grains of calomel to be taken at night, and a purgative draught on the following morning. The pain still continuing, to which was added a troublesome cough, I recommended a dentist to be sent for, who extracted the tooth very dexterously with a punch, but without any abatement of pain; which, indeed, increased regularly towards evening. The appetite now failed, which, with loss of rest, confinement, &c. reduced her very considerably. Having a very great repugnance to medicine, I could not prevail upon my patient to try any thing of that kind, with the exception of an occasional dose of calomel and salts, till February 26th, when she consented to try the carbonate of iron, in doses of half a drachm three times a-day, which caused so much nausea and griping, that, after persisting for one week, it was discontinued; but was resumed in a few days, in doses of two scruples, conjoined with a few grains of aromatic powder. In a few days it was increased to a drachm four times a-day, with a glass of port-wine after each dose, and continued regularly, with evident amendment, and with some days of total absence of pain. On the morning of the 16th, I was gratified to hear that she had had no pain whatever for the last week, and she requested to discontinue the powders, as they still disagreed with her bowels, though particular attention had been paid to them.

The cough is quite gone, her spirits are good, and the gum has lost all tenderness and pain upon mastication; the powders were consequently discontinued. E. S. SIMON SNOW, Surgeon.

With the following important communication, I have been favoured by J. Miles, Esq. surgeon, Throgmorton-street, London, detailing the very distressing case of his own lady.

*London, Throgmorton-street; Nov. 26, 1823.*

Dear Sir,—The profession, of which I am a member, must feel particularly obliged to you for promulgating your opinions of the efficacy of the ferri subcarbonas in neuralgic affections. Individually, I feel truly thankful for the benefit a valuable wife has received from the use of this important tonic, administered in the manner so ably recommended by you, in a decided case of tic douloureux, affecting the facial nerves of the left side: and, perhaps, it is an unusual circumstance that, during the whole course, menstruation has been suspended, not as a consequence of utero-gestation, though I suspected such was the case when the iron was first prescribed. I would beg briefly to relate the case, to show its apparent cause, its violence, and the superior efficacy of the iron over other remedies, which had been previously directed.

E. M., ætatis thirty, naturally healthy, but for some months has suffered from debility induced by protracted lactation, was attacked, in the early part of September, with most violent and intolerable pain of the facial nerves of the left side: this came on, and left her as suddenly, at various intervals; and, just at the time she would be expressing thankfulness at the subsidence of pain, the very motion of articulation, or even a stream of air coming on the face, would be sufficient to bring on a paroxysm of much violence. Various antispasmodics were internally resorted to; and a solution of the extract. belladon. with opium, was applied freely to the face, but followed by unimportant relief, and that of a very transitory kind. There being a partially-carious tooth in the lower jaw of the affected side, I was willing to suppose that had something to do with the keeping up the susceptibility to pain: this was consequently removed, with an aggravation of all my patient's sufferings. My friend, Mr. Thomas Bell, of Guy's Hospital, who extracted the tooth, recommended the trial of fifteen grains of the ferri subcarbonas three times a-day: in about three days, it certainly mitigated the urgency of the pain; but, in consequence of her respiration becoming much impeded, which before was perfectly uninterrupted, the iron was suspended. I had no sooner done so, than the breathing became easy; but the dreadful pain recurred with twofold violence. In this state of things, two drachms of the extract. cinchonæ were given, in divided doses, every twenty-four hours; and, before six drachms had been taken, some improvement was obvious. This plan was, without interruption, continued for a fortnight, with (at times) but comparatively slight pain. Occasionally, however, even at this period, the pain returned with so much violence, that it appeared to defy the aid and power of medicine. Under these very distressing circumstances, for they were truly so to me, as well as to the patient, I determined again on giving the iron in an increased dose: half a drachm was consequently administered three times a-day, with a vegetable infusion. After a lapse of three days, the happiest effects ensued. My patient has now taken the iron for three weeks: she has had no pain since the first three days; and, so highly important does she consider the absolute power of the medicine, that its continuance is strongly and most ardently solicited.

It would certainly appear that the bark was really important in the above case, inasmuch as it prepared the system for the more powerful tonic. The value of the ferri subcarbonas, however, has been so strikingly exemplified, that I could not refrain from making you acquainted with the result, in what I conceive to be so well-marked a case; and I really believe that, in all neuralgic affections, unconnected with derangement of the digestive organs, (for some cases of this disease certainly depend on, or are intimately connected with, such derangement,) I think the iron, administered in the manner so judiciously recommended by you, is the only medicine on which we can depend.

I am, dear Sir, your obliged servant,

S. MILES, Surgeon.

***To B. Hutchinson, Esq. Southwell.***

My ingenious friend, Dr. Marshall Hall, of Nottingham, has favoured me with the following very brief history:

*Nottingham; Nov.* 13, 1823.

My dear Sir,—Allow me to state to you, that, about a month ago, I was consulted by the lady in this neighbourhood, (whose case I briefly mentioned to you some few days back,) respecting the safety of the carbonate of iron in her distressing malady, which I shall take the liberty of shortly relating to you; this remedy having been recommended to her by Mr. Williams, surgeon, of this place.

The patient had suffered two attacks of paralysis, and after the last had been afflicted with the most excruciating cramps of the muscles of the legs; her nights' repose being much broken by her very severe sufferings.

Taking every circumstance of the case into consideration, I had no hesitation in giving it as my opinion that the remedy was perfectly safe. It was tried accordingly, and certainly with the most marked beneficial effects. The cramps ceased from the first or second day; and the general health of the lady, her spirits, and appearance, were remarkably improved. The relief appears to be permanent, having now suffered no spasms for five weeks.

Believe me, my dear Sir, ever most truly yours,

MARSHALL HALL, M.D. &c.

*To B. Hutchinson, Esq. Southwell.*

In a future communication, I shall take the liberty of offering to the perusal of your numerous correspondents some few of the results of my own experience, in treating the different modifications of neuralgia. I have, of late, had the pleasure of narrating what, in my estimation, is of much greater importance,—the experience of some among the most respectable and eminent of our practitioners.

It will not, I trust, be thought irrelevant to remark, on this occasion, that, in your valuable Journal of November last, I took the liberty of suggesting to your readers a caution against the employment of large doses of the carbonate of iron in the advanced stages of utero-gestation. In the last Number that has been published of an useful little periodical work, "The Medical Intelligencer," (the discontinuance of which I very much lament,) the ingenious editor has given the following commentary on my observations, to which I shall beg leave to make a very brief reply:—"In a short article, Mr. Hutchinson, of Southwell, cautions practitioners against the use of large doses of the carbonate of iron, in neuralgic diseases occurring during a state of utero-gestation; abortion, he thinks, being the most probable effect of so injudicious and hazardous a practice. Mr. Hutchinson states that he is induced to offer this remark and caution, in consequence of a lady, in the immediate neighbourhood of Southwell, who had been long affected with neuralgia faciei, having been recommended, by an eminent physician and a surgeon, to make use of Mr. Hutchinson's plan of treatment with the iron, in the latter months of her pregnancy; and the consequence being, as might easily be anticipated, abortion of a dangerous character.

"Now, we confess, we cannot see any reason for branding those gentlemen, who have been induced to exhibit the ferri subcarbonas to pregnant females labouring under neuralgia, as the followers of an injudicious and hazardous practice. We should be the last in the world to recommend any hazardous expedient; but we must own that we are somewhat sceptical regarding the *fancied* power of this metal as an abortive, to the extent which Mr. Hutchinson seems to imagine: and, even were it to act as such, we can see no reason why the resulting abortion ought readily to be anticipated as one of a peculiarly dangerous character. One solitary case of abortion, occurring during the administration of the iron, is perfectly insufficient to serve as the basis of these sweeping generalizations. Mr. Hutchinson is probably, however, possessed of others: if so, he would do a public service by stating them to the profession."

I beg to state, that my reply to the above observations was transmitted to the editor of the work alluded to, immediately after I had read the remarks; and the Medical Intelligencer being from that time suppressed, I have not enjoyed an opportunity, (owing to a dangerous and long-continued indisposition,) hitherto of explaining rather more fully the cause of my offering that caution to my brethren.

The absolutely *real*, and not *fancied*, power of the carbonate of iron, in producing abortions of a most perilous character, rests not on one, two, or three examples in the practice of well-experienced and highly skilful surgeons: the particulars of these cases shall be given in a future Number of your Journal, and that at no distant period. I shall beg, however, to remark, that even *one* dangerous case occurring from the exhibition of a medicine, whose *modus operandi* and influence over the uterine functions are well known and acknowledged, would act as a certain barrier against a repetition of a similar experiment.

Allow me to conclude by assuring your highly respectable readers, in the language which I addressed to the editor of the Medical Intelligencer, that, whenever I assume the privilege of troubling them with a perusal of the results of my practical observations, nothing in the shape of illusive hypothesis or theory shall ever tempt me to deviate from the true and unerring path of positive facts, deduced from ample and convincing experience. B. H.

*Southwell; Feb.* 14, 1824.

*Postscript.*—I have this moment had the pleasure of reading your last Number, containing a case of Cephalalgia, by Mr. Bushell, in which the sulphate of quinine was employed successfully. The treatment of the case, and its accurate description, are equally creditable to Mr. B.; but I will trouble you with the observations, which I will thank you to insert in your ensuing Journal, that many examples of similar derangements of the sentient powers have fallen under my notice and management during the course of the last two or three years. These cephalalgic complaints I have always been in the habit of considering as sympathetic, merely, of considerable disturbance of the chylopoietic functions; and my practice has been directed in consonance with these views. I have bled with freedom, and evacuated the bowels with calomel and jalap to a considerable extent; after which, small doses of the arsenical solution have seldom failed to effect the happy removal of the disease. I have never considered the sympathetic affections described by Mr. B. as genuine and legitimate cases of tic douloureux, and have, consequently, never treated them as such. B. H.

*Southwell; March 1st*, 1824.

